# A Fault Diagnosis Strategy for Analog Circuits with Limited Samples Based on the Combination of the Transformer and Generative Models

**DOI:** 10.3390/s23229125

**Published:** 2023-11-11

**Authors:** Zhen Jia, Qiqi Yang, Yang Li, Siyu Wang, Peng Xu, Zhenbao Liu

**Affiliations:** 1 School of Mechanical and Electrical Engineering, Xi’an University of Architecture and Technology, Xi’an 710055, China; yqq@xauat.edu.cn (Q.Y.); wangsiyu1@xauat.edu.cn (S.W.); xupeng139@xauat.edu.cn (P.X.); 2 School of Civil Aviation, Northwestern Polytechnical University, Xi’an 710072, China; yangli1001@mail.nwpu.edu.cn (Y.L.); liuzhenbao@nwpu.edu.cn (Z.L.)

**Keywords:** fault diagnosis, analog circuit, data generation, DCGAN, transformer

## Abstract

As a pivotal integral component within electronic systems, analog circuits are of paramount importance for the timely detection and precise diagnosis of their faults. However, the objective reality of limited fault samples in operational devices with analog circuitry poses challenges to the direct applicability of existing diagnostic methods. This study proposes an innovative approach for fault diagnosis in analog circuits by integrating deep convolutional generative adversarial networks (DCGANs) with the Transformer architecture, addressing the problem of insufficient fault samples affecting diagnostic performance. Firstly, the employment of the continuous wavelet transform in combination with Morlet wavelet basis functions serves as a means to derive time–frequency images, enhancing fault feature recognition while converting time-domain signals into time–frequency representations. Furthermore, the augmentation of datasets utilizing deep convolutional GANs is employed to generate synthetic time–frequency signals from existing fault data. The Transformer-based fault diagnosis model was trained using a mixture of original signals and generated signals, and the model was subsequently tested. Through experiments involving single and multiple fault scenarios in three simulated circuits, a comparative analysis of the proposed approach was conducted with a number of established benchmark methods, and its effectiveness in various scenarios was evaluated. In addition, the ability of the proposed fault diagnosis technique was investigated in the presence of limited fault data samples. The outcome reveals that the proposed diagnostic method exhibits a consistently high overall accuracy of over 96% in diverse test scenarios. Moreover, it delivers satisfactory performance even when real sample sizes are as small as 150 instances in various fault categories.

## 1. Introduction

The utilization of analog circuits is prevalent throughout numerous electronic devices, such as communication equipment and control systems. Because their components have poor tolerance, they are more susceptible to interference and influence. According to relevant surveys, approximately 80% of all electronic circuits employed within electronic equipment are digital circuits. While digital circuits account for the majority of electronic circuits, analog circuits continue to make up a small portion, around 20% of the total, resulting in excess of 80% of system faults [1]. In analog circuit faults, complex faults can directly cause the system to malfunction and are easily detected. Soft faults are mainly caused by abnormal changes in resistance, capacitance, and inductance parameters, and the measurement is complex and challenging [2]. Since analog circuits are composed of nonlinear and fault-tolerant components, the insufficient number of measurable nodes and measurement uncertainty make fault diagnosis complex. Therefore, realizing soft fault diagnosis at the component level is still challenging. Over the past few decades, numerous scholars have delved into this field, proposing various types of fault diagnostic approaches [3,4,5,6]. The field of artificial intelligence has experienced remarkable advancement in recent years, enabling the formulation and successful application of various deep learning models for fault diagnosis due to their excellent independent feature extraction capabilities and outstanding complex process generalization capabilities [7,8]. Recently, a deep neural network islanding detection technique based on statistical features was proposed in reference [9], and non-islanding disturbances were classified for hybrid systems based on synchronous and inverter distributed generators, which is a highlight in the field of fault diagnosis. The authors of [10] propose a novel technique for analog circuit fault detection using the application of image recognition, converting the power spectral density of the output signal into a two-dimensional image and inputting it into a deep convolutional neural network to achieve image classification and achieve the purpose of fault detection. Yu et al. [11] conducted research on a novel method for fault detection and diagnosis, which is predicated on a fusion of the firefly algorithm, tent chaos mapping, and extreme learning machine. The efficacy of this approach is exceptional in its ability to generalize well in the realm of fault diagnosis. Liu et al. [12] proposes a fault diagnosis method based on vibration sensor in ellipsoidal-ARTMAP network and differential evolution algorithm.

The remarkable diagnostic performance exhibited by many deep learning models is inherently tied to the acquisition of sufficient and uniformly distributed data samples. Nevertheless, in practical applications, the procurement of sufficient fault samples proves to be a formidable task owing to various reasons such as costs and safety concerns. When problems such as a small number of samples or different data distributions under different working conditions occur, the impact of a particular factor on the efficacy and precision of the neural network-based fault diagnosis system will be significant. The deep learning model requires much data support to exert its powerful capabilities in data modeling and classification identification. It also requires sufficient data coverage under different working conditions to ensure the capability of a deep learning model to extend its knowledge learned and evolve to novel situations, which is evaluated in [13]. When the limited amount of data is utilized directly for deep learning training, it will lead to extremely serious overfitting, especially not conducive to fault diagnosis. Effective training of deep learning models with small sample datasets requires preprocessing of the data, which is pivotal to achieving optimal training outcomes. The core of small sample fault diagnosis is to resolve the issue of an insufficient number of samples. In the field of fault diagnosis research, using original samples to expand the amount of data or improve data quality is an effective solution. The existing main methods to expand the target sample size are the Synthetic Minority Oversampling Technique (SMOTE), variational autoencoder (VAE) [14], autoregressive model [15], and generative adversarial network (GAN) [16,17]. Chawla et al. [18] introduced the SMOTE technique, which increases the quantity of samples available for analysis by finding similar samples near the minority class samples and synthesizing new small samples. However, it should be noted that when the samples are distributed on the edge of the classification, the samples synthesized by the SMOTE method classification boundaries may be blurred. Autoregressive models can perform density estimation on sequence data well, but their computational load is much larger than that of VAE and GAN. The VAE method is a commonly used generative model. Through the hidden features (low-dimensional hidden variables) learned in the input data, these hidden features are sampled and reconstructed, and finally, new data similar to the input data are generated, such as in reference [19], which introduces a variational autoencoder (VAE) into the fault diagnosis, facilitating data augmentation through the generation of vibration signals. Subsequently, an enhanced fault diagnosis method is proposed, wherein a convolutional neural network is combined with the aforementioned approach to realize improved accuracy and performance. Although the VAE method is trained and the training process is relatively stable, the generated pictures are relatively blurred, and the generated samples lack the capability to capture comprehensive fault information. In addition, the authors of [20] designed an adversarial transfer network comprising multiple scales based on an attention mechanism that automatically distinguishes various bearing fault states. The TrAdaBoost method is different from other methods of expanding the target sample size. It uses auxiliary datasets for training and applies them to the training of classifiers through weight adjustment. Xiao et al. [21] used CNN as a classifier, combined with the method of assigning weights to joint training datasets and the weight update of the TrAdaBoost model to achieve accurate fault diagnosis with high precision for small samples. The TrAdaBoost method is flexible and easy to operate when combined with other classifiers but requires a suitable and similar auxiliary dataset, which is difficult to obtain. In addition, classification difficulty increases when there is noise in the dataset itself.

In 2014, Goodfellow et al. [22] summarized the characteristics of the generative and discriminative models in machine learning and proposed a creative network model-generated confrontation network (GAN). A novel deep learning approach has emerged that can be utilized for the purpose of data augmentation, data generation, data modeling, and other fields. Traditional GAN uses Jensen–Shannon divergence (JS divergence) to quantify the degree of congruence between false images and authentic images. With the in-depth research on the GAN network, researchers found that the network is prone to problems such as network collapse and gradient explosion during the training process, which makes the GAN network challenging to train. In order to address the aforementioned issues, more and more variant algorithms have been proposed. Least Squares Generative Adversarial Networks (LSGAN) [23] are designed to enhance the objective function of a GAN network, specifically the discriminator, by replacing the cross-entropy loss function with a least squares loss function, making the transfer of gradients more effective and the model’s training process more stable. Liu et al. [24] advanced a technique that amalgamates variational autoencoding and GAN to glean precise features of real data samples in the context of data scarcity. Wasserstein GAN [25,26] effectively substitutes the Kullback–Leibler (KL) divergence and Jensen–Shannon (JS) divergence with the Wasserstein distance in order to accurately measure the distance between the generated distribution and the actual distribution and make the gradient calculation of the generator more accurate. The conditional generative adversarial network (CGAN) [27] proposes adding conditional information to GAN’s network structure so that GAN can accurately train and generate images of various categories in one model simultaneously and according to different datasets and generation requirements. Similarly, Dixit et al. [28] used CGAN to generate vibration signals of rotating machines. Then, they used auxiliary classifiers as GAN discriminators in these signals and performed meta-learning to improve the generalization of classifiers’ capabilities for better fault diagnosis. References [29,30,31] go further based on CGAN by adding classifiers and clusters inside GAN to partially or entirely obtain condition information. Then, they conduct model training similar to CGAN, thus partially or entirely removing the limitations of label information on CGAN training. The authors of [32] propose the use of a temporal generative adversarial network, coupled with an efficient network model, to implement a transfer learning method, displaying excellent levels of effectiveness, reliability, and generalization performance. Deep Regret Analytic Generative Adversarial Networks (DRAGAN) [33] introduce the no-regret algorithm in game theory and transform its loss function to solve the GAN collapse problem. The authors of [34] incorporate a discriminator into its network design to resolve the issue of restricted variety within the generated samples. Rad-ford et al. [35] proposed the introduction of a convolutional neural network in place of the original fully connected network within the architecture of the GAN to develop the deep convolutional generative adversarial network (DCGAN), which significantly improved the generation and generalization capabilities of the GAN network model and provided new ideas for the development of GAN networks. The authors of [36] have employed DCGAN to convert one-dimensional vibration data into grayscale images, thereby increasing the availability of fault data samples and resolving the issue of inadequate data samples.

The Transformer model [37] was proposed by Google in 2017. One of the most notable characteristics of this particular model is its departure from network structures typically utilized in RNN and CNN. The Transformer model has gained significant prominence in the realm of machine translation due to its remarkable capabilities. Recently, there has been a growing interest in applying the Transformer model to various fields, such as sequence data prediction, target detection, and image classification, leading to promising outcomes [38,39,40]. The Swin Transformer [41], proposed by Microsoft Research Asia, has attained superior performance in multiple vision tasks, indicating its state-of-the-art nature. As the number of network layers increases, the attention mechanism may collapse, resulting in a decrease in Transformer model performance rather than an increase. To address this challenge, Touvron et al. [42] conducted research based on the ViT model and designed a layer scale structure that can also converge when the network is deepened, solving the problem of variance amplification in the residual connection process. A proposed method for fault diagnosis in rotating machinery utilizes intelligent feature self-extraction and transformer neural network techniques [43]. The multi-scale SinGAN model [44] is especially adept at generating high-quality kurtosis map images, which can play a pivotal role in effectively training complex machine learning models.

The methods proposed in the above literature have shown good learning capabilities and transfer effects in various unsupervised cross-domain fault diagnosis tasks, providing an essential reference for subsequent research in this field. To enhance the precision, stability, and broad applicability of diagnostic assessments, a set of innovative enhancements have been proposed.

Contributions: Our approach is made possible by the following technical contributions:

(i) Using continuous wavelet transform (CWT) can enhance the processing of analog circuit signals in the time–frequency domain, efficiently converting one-dimensional signals into two-dimensional time–frequency images that are advantageous for fault feature selection, leading to improved training speeds and enhanced efficiency;
(ii) A data augmentation approach based on DCGAN has been designed, using a generative model to generate convincingly realistic new images, effectively amplifying the image set. The amplified dataset is used to support model training, improving its performance and generalization capabilities, with the aim of addressing the problem of overfitting commonly experienced with small image datasets;
(iii) A fast and high-precision diagnosis technology based on the Transformer model is proposed, which greatly shortens the training time and achieves a good diagnosis accuracy–speed trade-off.

The rest of the content is organized as follows: In Section 2, the theoretical background and the proposed method are outlined. Section 3 illustrates the validation of the proposed method by detailing the selection of three experimental circuits and the construction and preparation of the fault dataset. Section 4 delves into the experimental outcomes and analysis. The final section, Section 5, provides a comprehensive summation of the complete paper.

## 2. Related Theories

### 2.1. Continuous Wavelet Transform

Continuous wavelet transform (CWT) can display the time–frequency characteristics of the vibration signal on the image. For a mother wavelet function, $\varphi (t)\in L^{2}(R)$, the Fourier transform satisfies the following equation:

(1) $$\int_{-\infty }^{+\infty }\frac{|\hat{\varphi }(\omega ){|}^{2}}{\omega }d\omega <\infty$$

where ω denotes frequency, and $\hat{\varphi }(\omega )$ denotes Fourier transform of φ(t). Stretch and translate to obtain a family of wavelet functions as follows:

(2) $$\varphi_{a,b}(t)=\frac{1}{\sqrt{a}}\varphi (\frac{t-b}{a})\;a,b\in R,a>0$$

where $t=at_{0}+b$, a is the scale factor, b is the displacement factor, and $\varphi_{a,b}(t)$ is the analysis wavelet. The scale factor determines the extent to which the wavelet function can be scaled in the frequency domain, while the displacement factor governs the extent to which the wavelet function can be displaced in the time domain. Combined with the above explanation, the *CWT* transform definition of any finite energy signal $x(t)\in L^{2}(R)$ is as follows:

(3) $$CWT_{x}(a,b)=[x(t),\varphi_{a,b}(t)]=\frac{1}{\sqrt{a}}\int_{-\infty }^{+\infty }x(t)\varphi^{*}(\frac{t-b}{a})dt$$

where $\varphi^{*}(\frac{t-b}{a})$ is the conjugate of $\varphi (\frac{t-b}{a})$; $CWT_{x}(a,b)$ is the scalar product of signal *x*(*t*) and wavelet $\varphi_{a,b}(t)$.

### 2.2. Deep Convolutional Generative Adversarial Networks

The concept of adversarial training for GAN networks stems from the field of game theory, and its main network model is composed of a set of generators built by deep networks and another set of discriminators built by deep networks. Deep convolutional generative adversarial networks are an innovation in the basic framework of GAN and are particularly suitable for processing two-dimensional data such as images and three-dimensional data [45]. Like GAN, the game comprises a generator and discriminator, which engage in competition through their respective operations and ultimately reach a state of Nash equilibrium [21].

**Figure 1:**
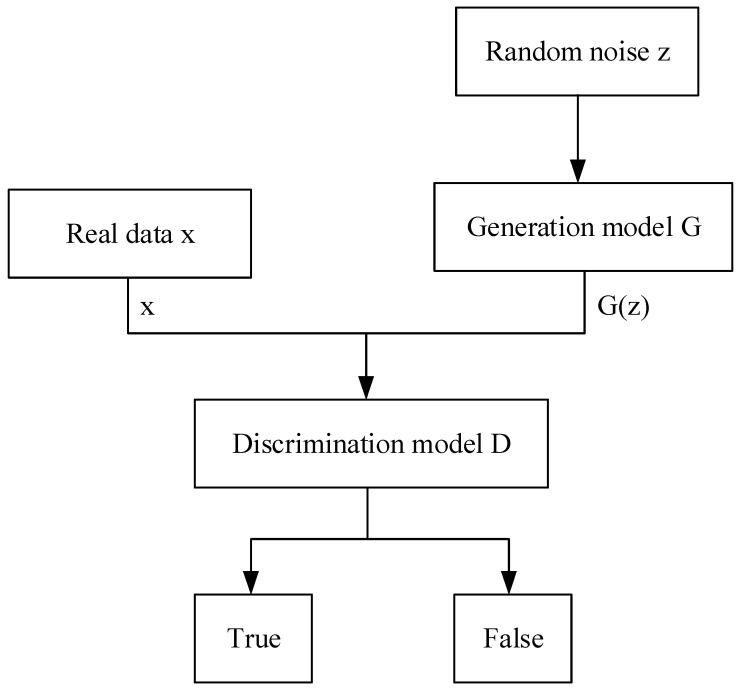
Basic structure of DCGAN.

The basic structure is shown in Figure 1. *z* is the input random noise, and *G* is the generator. G(z) represents the generated picture, *x* is the real data, and *D* is the discriminator. The random noise (*z*) is inputted into the generative model, and the model parameters are learned. In the generative model, it is gradually converted into the sample output close to the real data. And the discriminative model is responsible for evaluating whether the samples generated by the generator network are close to the real data. The training process for the generator and discriminator involves training them alternately, with the generator endeavoring to deceive the discriminator and the discriminator endeavoring to discern the difference between the generated samples and the authentic data. This process is maintained until the output of the generator network approximates the real data to a degree where the discriminator network cannot distinguish the samples.

Through a consistent process of learning and refining, the generator enhances its capacity to produce sample data. Therefore, the training problem of *G* can be expressed as a value function V(D,G), which becomes a maximization and minimization problem of V(D,G). This can be expressed as follows:

(4) $$\underset{G}{\min}\;\underset{D}{\max}\;V(G,D)=E_{x∼P_{data}(x)}\left[\mathrm{lg}D(x)\right]+E_{z∼P_{z}}(z)\left[\mathrm{lg}(1-D(G(z)))\right]$$

where x is the input of the network comprises real images, D(x) represents the likelihood of real images, z denotes the level of noise present in the image inputted into the generator, G(z) symbolizes a fictitious image generated by the generator, and D(G(z)) signifies the probability of a false image.

Equation (4) is split into two loss function models. In the architecture of the discriminator model, the loss function is given by the following Equation (5):

(5) $$\underset{D}{\max}\;V(D,G)=E_{x∼data(x)}[\mathrm{lg}(D(x))]+E_{z∼P_{g}(z)}[\mathrm{lg}(1-D(G(z)))]$$

Among them, the first item on the equation is the real sample’s discrimination result, with the closer it is to 1 indicating a better performance. The second term refers to the newly generated sample’s discrimination result, which should be closer to 0 to achieve optimal performance.

Obtaining the generator loss function follows the same principle as the discriminator loss function and is calculated using Equation (6):

(6) $$\underset{D}{\min}\;V(D,G)=E_{z∼P_{g}(z)}[\mathrm{lg}(1-D(G(z)))]$$

In contrast to the discriminator, the generator only needs to approach 1 on the generated data.

The theoretical basis is analyzed as follows:

Starting from the objective function, since the value function V(D,G) is continuous, Equation (1) is written in calculus form to express the mathematical expectation as Equation (7):

(7) $$V(D,G)=\int_{-x}^{x}P_{data}(x)[\mathrm{lg}(D(x))]+P_{g}(z)[\mathrm{lg}(1-D(G(z)))]dz$$

Assuming that the data generated by the generator G(z) are x, the expressions for the noise point z and the differential dz are calculated as (8) and (9), respectively:

(8) $$x=G(z)\Rightarrow z=G^{-1}(x)$$

(9) $$dz=(G^{-1}{)}^{\prime }(x)dx$$

Upon the substitution of Equations (7) and (8) into Equation (9), a resultant expression is generated.

(10) $$V(G,D)=\int_{-x}^{x}P_{data}(x)[\mathrm{lg}D(x)]dx+\int_{-z}^{z}P_{z}(G^{-1}(X))[\mathrm{lg}(1-D(x))](G^{-1}{)}^{\prime }(x)dx$$

Define the generating distribution of the noise input *z* as $P_{g}(x)$, then

(11) $$P_{g}(x)=P_{z}(G^{-1}(x))(G^{-1}{)}^{\prime }(x)$$

where $P_{z}$ denotes the generating distribution, adding noise to the generator, and we obtain the following:

(12) $$V(D,G)=\int_{-x}^{x}P_{data}(x)[\mathrm{lg}D(x)]dx+\int_{-x}^{x}P_{g}(x)[\mathrm{lg}(1-D(x))]dx$$

Evaluating the maximum value of the function V(G,D), fixing *G*, and taking the partial derivative of *D* yields the following:

(13) $$D^{*}(x)=\frac{P_{data}^{}(x)}{P_{data}(x)+P_{g}(x)}$$

As can be seen from the expression of $D^{*}(x)$, consistency in the distribution of generated virtual sample data and real data is achieved when the latter are deemed congruent and similar; thus, immediately, $P_{data}\left(x\right)=P_{g}\left(x\right)$. The condition is satisfied under this condition $D^{*}(x)=0.5$; at this time, the *D* network has been unable to judge the truth or falsity of the sample data generated by the G network; that is, *D* has achieved the optimal solution. If and only then $P_{data}\left(x\right)=P_{g}\left(x\right)$, the maximization and minimization problem of V(D,G) has a globally optimal solution; that is, it reaches the Nash equilibrium state. It is possible to stop the training procedure at this point since model *G* has mastered the actual sample distribution, and the accuracy of model *D* has remained consistent at 50%.

### 2.3. Transformer

Transformer, a deep model widely used in natural language processing and image analysis, can efficiently process sequence data, capture features by modeling the dependencies inside the sequence, and improve computational efficiency without a loop structure. And its individual components are analyzed next.

#### 2.3.1. Attention Mechanism

**Figure 2:**
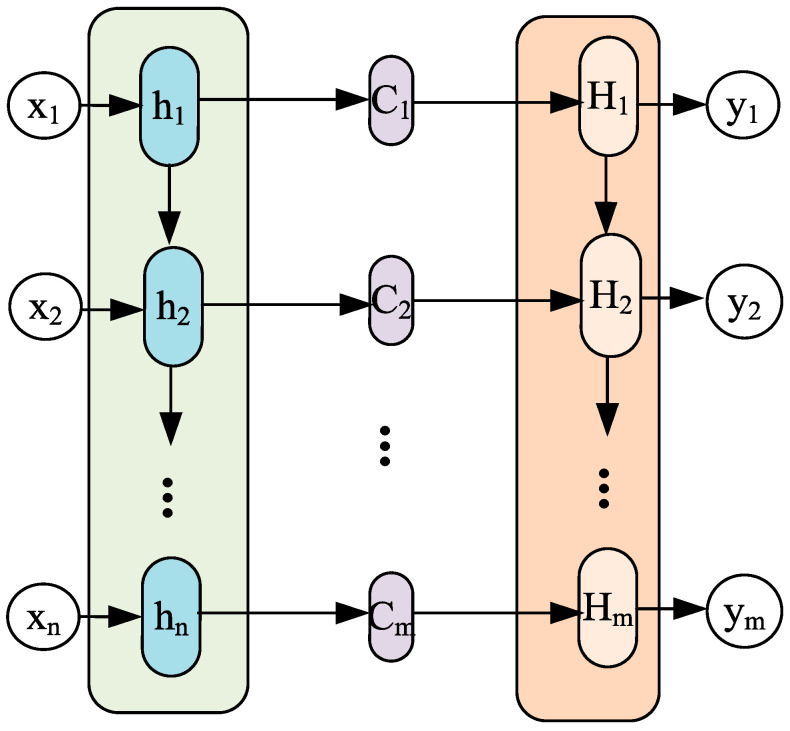
Encoder–decoder framework with attention mechanism.

The source of the attention module is to learn from human attention and intuitively explain it by the attention mechanism of human vision. Concretely, machine learning can be viewed as a mathematical computation of data attributes, which takes into account certain weights. These weights are attention. As a member of the attention module, self-attention can be used to calculate the interdependence between different regions inside the image. The common attention mechanism is soft attention (SA). The attention mechanism can be used in any model. This paper presents a novel technique, SA, which is grounded in the encoder–decoder framework. The encoder–decoder framework has gained widespread acceptance in natural language processing and time series prediction. As shown in Figure 2, the input sequence can be represented as $\left[x_{1},x_{2},\cdot \cdot \cdot ,x_{n}\right]$ and the output sequence as $\left[y_{1},y_{2},\cdot \cdot \cdot ,y_{m}\right]$, where the attention result $c_{i}$ is calculated by the following formula:

(14) $$c_{i}=\overset{n}{\underset{j=1}{\sum }}a_{ij}h_{j}$$

where n is the input data length of the coding layer; $h_{j}$ is the hidden layer state of the j input data in the coding layer; $a_{ij}$ represents the attention distribution coefficient of the j data in the encoding layer when the i value is output by the decoding layer. $a_{ij}$ is calculated as the following:

(15) $$a_{ij}=F(H_{i}h_{j})$$

where $H_{i}$ is the hidden layer state of the i data in the decoding layer, and F is a function. Calculate the similarity of $H_{i}$ and $h_{j}$, then the output of the function F is normalized by Softmax to obtain the attention.

**Figure 3:**
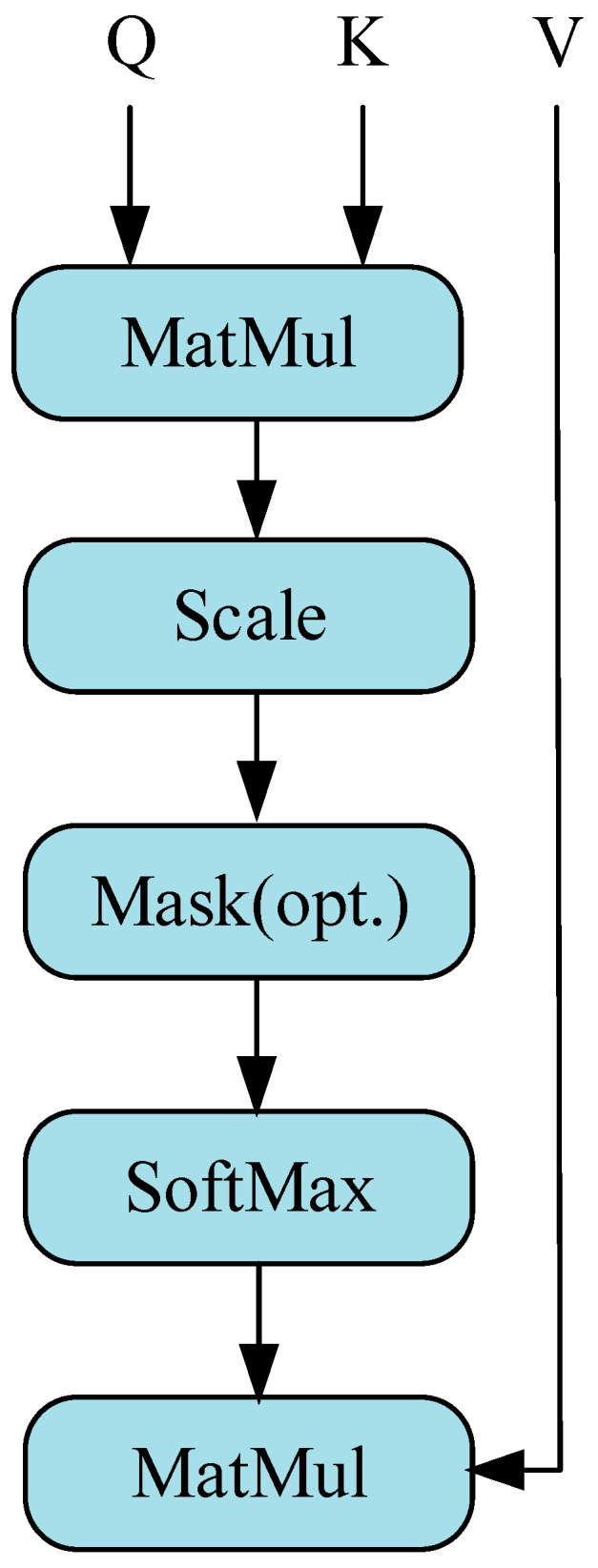
Self-attention with attention mechanism.

The scaled dot product attention used in ViT is shown in Figure 3. Denoted by $X\in R^{n\times d}$ a sequence of n elements is (*x*_1_, *x*_2_, *…*, *x_n_*), and d denotes the embedding dimension of each element. And through the interaction between Query and Key–Value pairs, the self-attention layer achieves dynamic aggregation of information, which is obtained as follows:

(16) $$Q=XW^{Q},K=XW^{K},V=XW^{V}$$

The calculation of scaling dot product attention proceeds in the following manner:

(17) $$(Q,K,V)=softmax(\frac{QK^{T}}{\sqrt{d_{z}}}{)}^{V}$$

where $QK^{T}$ is the attention score, and $d_{z}$ is the dimension of vectors *Q* and *K*. $\sqrt{d_{z}}$ is the scaling factor.

**Figure 4:**
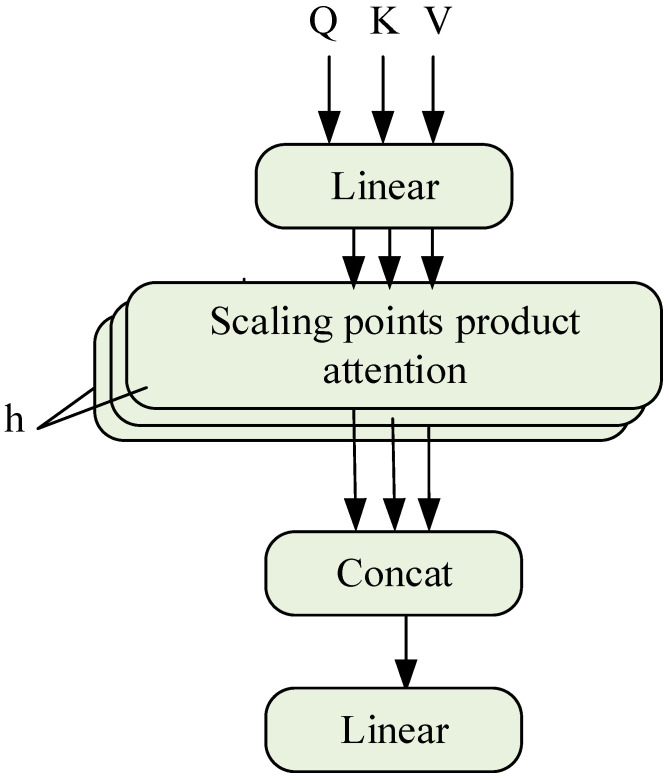
Multi-head self-attention.

Multi-head attention (MHSA) has multiple independent self-attention layers (heads), each with its own learnable weight matrix, and the calculation process is $W_{i}^{Q},W_{i}^{K},W_{i}^{V}$, as shown in Figure 4 and as shown in Equations (18)–(20):

(18) $$Q_{i}=XW_{i}^{Q},K_{i}=XW_{i}^{K},V_{i}=XW_{i}^{V}$$

(19) $$Z_{i}=Attention(Q_{i},K_{i}),i=1,2,\cdots ,h$$

(20) $$(Q,K,V)=Concat(Z_{0},Z_{1},\cdots ,Z_{h})W^{0}$$

where h refers to the number of attention heads of MHSA, $Z_{i}$ represents the output vector of each attention head, $W_{}^{0}$ is the output projection matrix, and $W_{i}^{Q},W_{i}^{K},W_{i}^{V}$ is the learnable weight matrix. $Q_{i},K_{i}$ and $V_{i}$ can be regarded as the split under different feature subspaces in single-head attention, and the correlation between features is extracted from multiple angles without increasing additional computational cost. Finally, the information extracted by each self-attention layer is merged to obtain more rich and more comprehensive feature information.

#### 2.3.2. Transformer Model

**Figure 5:**
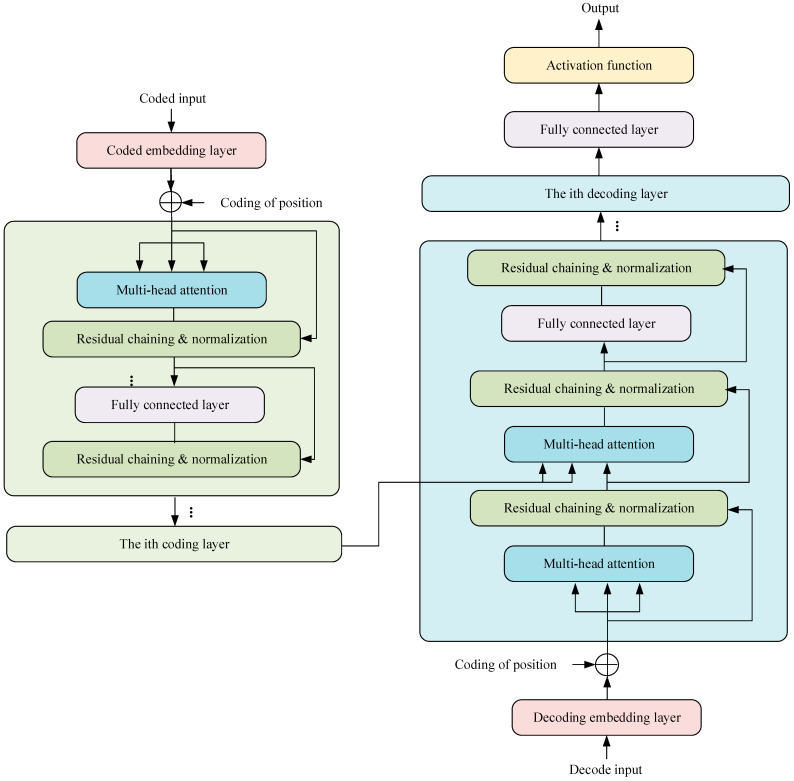
Transformer model framework.

The Transformer model, illustrated in Figure 5, consists of an encoding block and a decoding block. The contrast between the decoding layer and the encoding layer lies in the fact that each decoding layer comprises two multi-head attention layers. In the decoding layer, given its two multi-head attention layers, the initial attention layer mirrors that of the decoder layer. Correspondingly, the *K* and *V* fields of the second attention layer are the respective outputs of the decoding block. Lastly, the regularization layer furnishes the *Q* for the attention mechanism of this layer. In addition, the structure of the regularization layer in the Transformer is consistent, which is mainly composed of residual connections and regularization operations:

(21) $$norm_{cur}=Normalization(z,norm_{\mathrm{out}})$$

where *z* is the output of the attention or fully connected layer.

### 2.4. The Proposed Method

**Figure 6:**
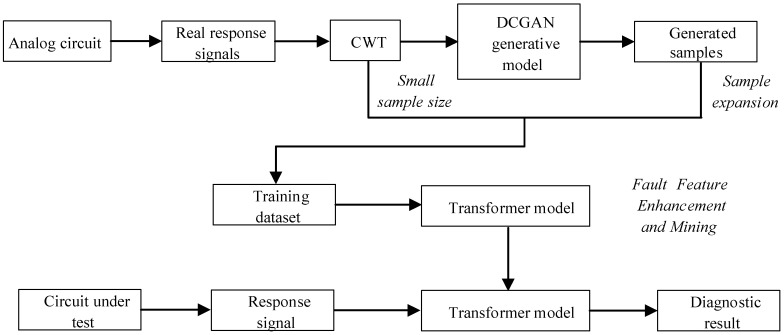
Flowchart of the proposed analog circuit small sample fault diagnosis scheme.

The first step involves collecting the real output voltage signal of the analog circuit. After that, the output signal’s time–frequency characteristics are extracted using the method of CWT. Then, the DCGAN generation model is trained by using the real-time–frequency feature graph, and a new time–frequency feature graph can be generated by inputting the real time–frequency feature graph into the trained DCGAN model. Mixing real time–frequency signals and generated time–frequency signals can be used to train the Transformer model, realize automatic mining and classification of time–frequency features, and finally complete fault diagnosis. The diagram representing the proposed diagnostic strategy’s workflow is displayed in Figure 6.

The model’s training procedure entails the following sequence of steps:

The first step is DCGAN training: the generator generates new sample data (fake sample) by constantly transforming random noise pictures conforming to a normal distribution. The discriminator network receives the fake sample and the real sample and determines whether the image data correspond to the output image by calculating the probability of the generator. The generator and discriminator engage in a continuous optimization process through adversarial confrontation until the loss function converges. At that point, the generator model is saved, and additional sample data are generated.

The next step involves utilizing the Transformer model to pre-train the newly generated sample data, adjusting the parameters until the model converges and reaches a better evaluation index, and saving the pre-trained model.

Thirdly, use a Transformer to conduct iterative training and testing on the original sample data until the training converges. In the event that convergence has not been achieved, the learning rate, as well as the dimensions and amount of convolutional layers, will be adjusted until convergence is attained.

## 3. Analog Circuit Experiment and Fault Setting

This section primarily covers topics related to analog circuit examples, fault configuration and data acquisition, signal preprocessing methods, and diagnostic model parameter settings. These are preparatory steps for the subsequent presentation and analysis of the experimental results in Section 4.

In the process of operating analog circuits, the performance of capacitors, resistors, and other components inevitably deteriorates. Deviations in the actual output signal value from the theoretical value may occur due to various factors, resulting in an uneven result. In general, the device has a normal value for its operation X, tolerance α, and threshold β. The device can still work normally under the condition of working within the tolerance range. If the device works beyond the tolerance range but does not exceed the threshold range, the circuit has a soft fault. In the event that the device parameters exceed the pre-established threshold, the circuit will cease functioning altogether. To ensure the efficacy of the suggested algorithm, three circuits, including the Sallen–Key bandpass filter circuit, the Biquad low-pass filter circuit, and the Thomas filter circuit, were subjected to experimental verification, as illustrated in the accompanying figure.

### 3.1. Fault Settings

**Figure 7:**
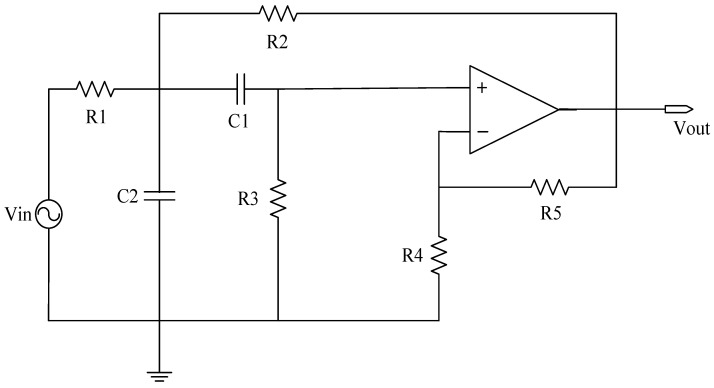
The circuit schematic of Sallen–Key bandpass filter.

**Figure 8:**
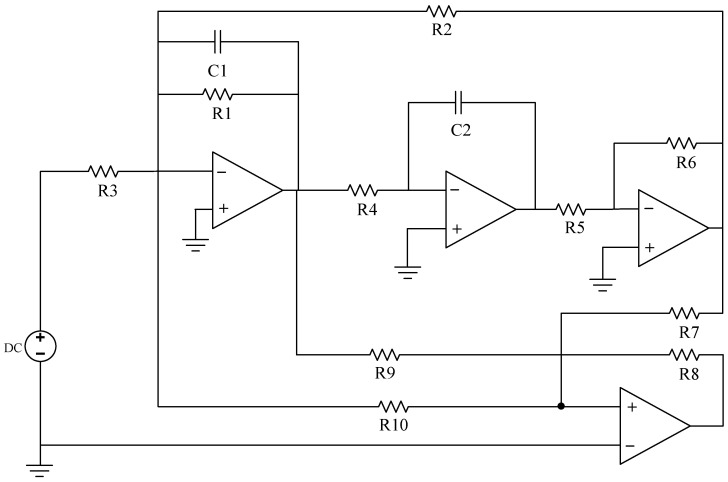
The circuit schematic of Biquad low-pass filter circuit.

**Figure 9:**
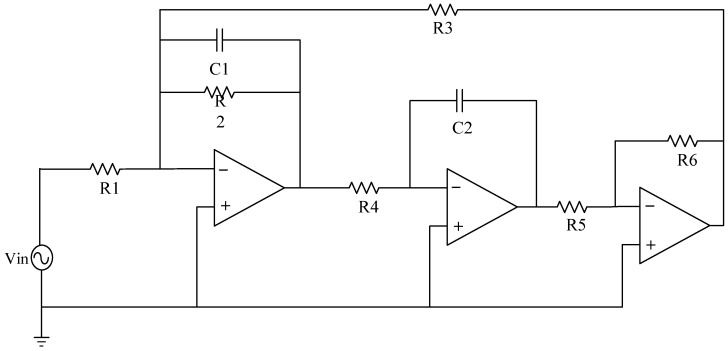
The circuit schematic of Thomas filter circuit.

In the field of circuit fault diagnosis, the selection of faulty components is typically determined based on an analysis of which components have a significant impact on the input. This determination is generally guided by the circuit’s transfer function. The specific approach involves conducting sensitivity analysis using circuit simulation software to identify components that have a substantial influence on the circuit’s output. These components are then considered as potential candidates for faults, thus laying the groundwork for subsequent research. If only one device in the analog circuit has a soft fault, it is called a single fault, and its fault settings are depicted in Table 1 and Table 2. In the event of multiple components experiencing simultaneous failures, it is referred to as a compound fault, wherein the fault signal manifests similar properties to those observed with a single fault. Although the incidence is low, its low feature recognition rate and many fault modes cannot be ignored, and its fault settings are depicted in Table 3. In an analog circuit, the component that has a significant impact on the output of the circuit is referred to as the sensitive element. The Sallen–Key bandpass filter circuit, depicted in Figure 7, exhibits a number of sensitive components, including C1, C2, R2, and R3. The Biquad low-pass filter circuit illustrated in Figure 8 features a number of vulnerable components, including C1, C2, R1, R2, R3, and R4. Lastly, the Thomas filter circuit, shown in Figure 9, possesses numerous sensitive components, including C1, C2, R3, R4, and R5. To conduct an analog circuit fault test, it is recommended to adjust the device parameter upwards (↑) or downwards (↓) by a specified amount that exceeds the standard value. This measure aims to identify any potential issues within the circuit. The tolerance of the resistance and capacitance of the circuit are 5% and 10%, respectively, and the fault threshold is set to 30%. Specifically, the range of resistance failure in the circuit is encompassed within the limits of [70% X, 95% X]∪[105% X, 130% X]. Likewise, the range of capacitor failure in the circuit is restricted within the bounds of [70% X, 90% X]∪[110% X, 130% X], where X denotes the nominal value of the component.

#### 3.1.1. Sallen–Key Bandpass Filter Circuit

The single fault set has been established to encompass the states {C1↑, C1↓, C2↑, C2↓, R2↑, R2↓, R3↑, R3↓, NF} in a total of nine distinct configurations, as per 0.

Combining the single fault in Table 1 in pairs to form a compound fault, the parameters of the circuit components remain unchanged, and the complex fault set is set to {C1↑C2↑, C1↓C2↓, C2↑R2↑, R2↓R3↑, R2↓R3↓C1↑, R3↑C1↑C2↓, NF}, which can be in eight states.

#### 3.1.2. Biquad Low-Pass Filter Circuit

Like the Sallen–Key bandpass filter circuit, in the Biquad low-pass filter circuit, the normal values of capacitors C1 and C2 are both 5.00 nF, and the fault value range is [3.50 nF, 4.50 nF]∪[5.50 nF, 6.50 nF]; the normal value of R1, R2 and R3 is 6.20 kΩ, and the fault range is [4.34 kΩ, 5.89 kΩ]∪[6.51 kΩ, 8.06 kΩ]. As previously described, the single fault set is constructed as {C1↑, C1↓, C2↑, C2↓, R1↑, R1↓, R2↑, R2↓, R3↑, R3↓, R4↑, R4↓, NF}, with a total of 13 states, and the composite fault set can be created in eight states: {C1↑C2↑, C1↓C2↓, C2↑R2↑, R2↑R5↑, R1↑R2↑C1↑, R3↑C1↑C2↓, NF}, with a total of eight states.

#### 3.1.3. Thomas Filter Circuit

The single fault set is constructed as {C1↑, C1↓, C2↑, C2↓, R3↑, R3↓, R4↑, R4↓, R5↑, R5↓, NF}, with a total of 11 states. The specific settings are shown in Table 2.

The composite fault set is created in eight states: {C1↑C2↑, C1↓C2↓, C2↑R3↑, R3↑R5↑, R3↑R5↑C1↑, R3↑C1↑C2↓, NF}, with a total of eight states. And Table 3 lists the settings.

### 3.2. Signal Preprocessing

**Figure 10:**
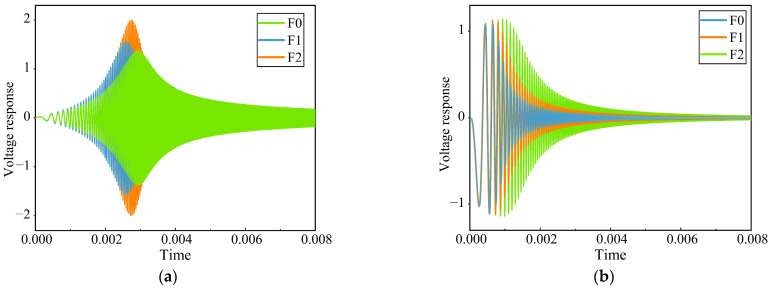
The response of analog circuits under different fault states. (**a**) Sallen–Key bandpass filter circuit. (**b**) Thomas filter circuit.

In order to enhance the training capacity of the model and minimize the number of computations required, it is imperative to undertake the process of data preprocessing prior to its collection. As shown in Figure 10, the output voltage signals of the Sallen–Key bandpass filter circuit and the Thomas filter circuit under partial fault conditions are shown.

A. Standardized processing

To further standardize the collected data and better train the neural network model, the data of each sample are standardized. The standardization method adopted in this paper is min-max standardization. Its mathematical expression is as follows:

(22) $$X=\left|x_{i}\right|=\frac{x_{i}-x_{min}}{x_{max}-x_{min}}$$

B. Spectrogram conversion

**Figure 11:**
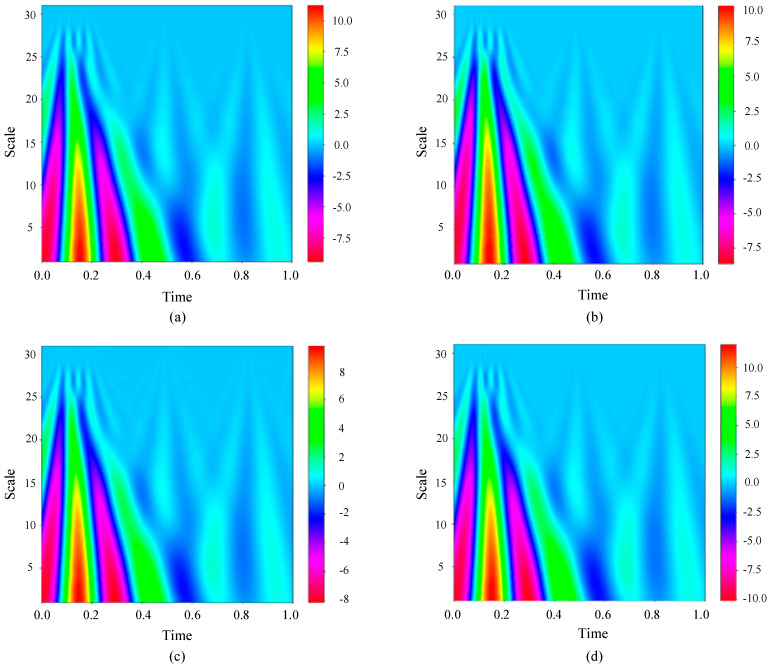
Continuous wavelet transform results of Sallen–Key bandpass filter circuit response under different fault states. (**a**) F0. (**b**) F2. (**c**) F3. (**d**) F5.

Consider the Sallen–Key bandpass filter circuit as an example. When continuous wavelet transform processing is applied to the output signal, the resulting graph can be seen in Figure 11.

Upon reviewing Figure 11, it is evident that the transformations resulting from various fault categories display subtle distinctions in the accompanying images. Therefore, manual classification is difficult, and automatic recognition technology is very necessary to explore the differences between different faults.

C. Dataset partitioning

A total of 300 samples were collected for each fault type in both single-fault and multi-fault circuit cases. The samples were subjected to the aforementioned pretreatment procedure. A training dataset of 300 samples was assembled for each fault type, as the size of the data was determined based on a combination of real samples and generated samples with varying mixing ratios. A crucial aspect of the training process is to establish the ratio of real samples to generated samples in order to optimize the performance of the fault diagnosis model.

### 3.3. Fault Signal Collection

**Figure 12:**
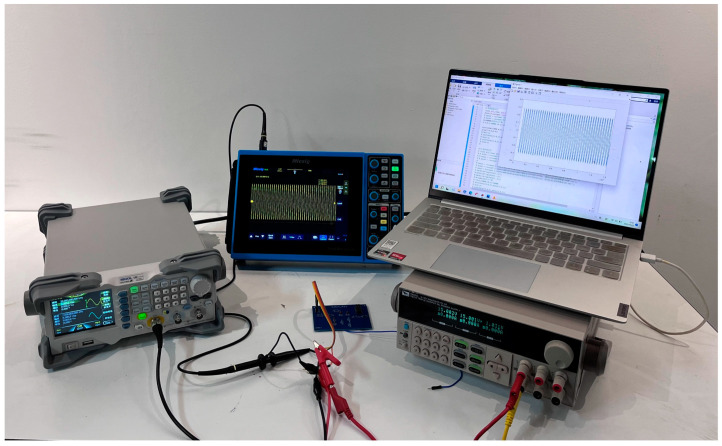
Experimental platform built.

The experimental platform depicted in Figure 12 demonstrates a viable method for data acquisition. The equipment utilized incorporates an Agilent 33250 arbitrary waveform generator, Agilent 54853 digital oscilloscope, power supply, LabVIEW_2023 Q1 software, National Instruments 1042q data acquisition module, and the circuit under test (CUT). The arbitrary waveform generator creates a sinusoidal scanning excitation signal, which is then recorded using LabVIEW software. The test circuit’s output signal is then collected through the input of the sinusoidal excitation signal under various faults. Since the output signal is periodic, data analysis will involve collecting the signal for 50 cycles and dividing it into 50 samples. Specifically, to obtain accurate data, the first 10ms of the output signal will be collected with the sampling frequency set at 60 kHz and the sampling point set at 600. Each fault type and non-fault state will be assigned 500 labeled samples. Subsequently, the samples will be normalized and divided evenly into five separate subsets for five-fold cross-validation.

### 3.4. Parameter Settings Involved in the Proposed Diagnostic Strategy

**Figure 13:**
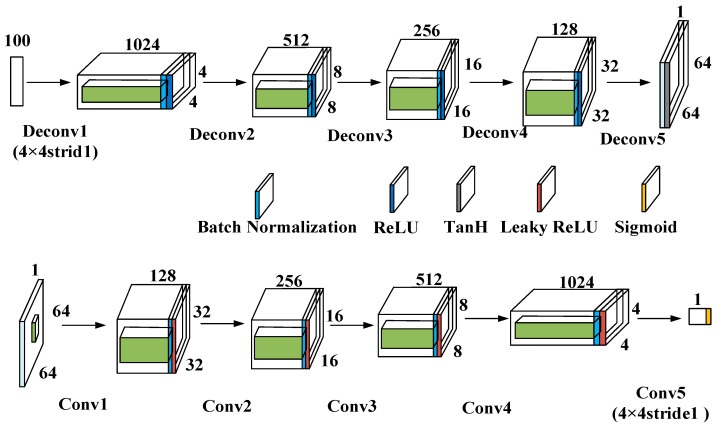
DCGAN network structure.

As depicted in Figure 13, a pragmatic, experimental platform has been developed for data acquisition. It encompasses an Agilent 54853 digital oscilloscope, a circuit under test (CUT), LabVIEW software, an Agilent 33250 arbitrary waveform generator, a National Instruments 1042q data acquisition module, and a power supply.

The DCGAN data generation model comprises a generator and a discriminator, both of which are components of the DCGAN architecture.

The DCGAN generator uses five transposed convolutional layers to upsample a 100-dimensional noise vector. The final output is a 64 × 64 × 1 image. Each convolutional layer, when transposed, has a stride of 2 and a 4 × 4 kernel size. The output dimensions of each transposed convolutional layer are as follows: 1024 × 4 × 4, 512 × 8 × 8, 256 × 16 × 16, 128 × 32 × 32, 1 × 64 × 64.

The architecture of the DCGAN discriminator mirrors that of the generator in a symmetric manner. It takes 64 × 64 × 1 images as input and uses five convolutional layers for feature extraction, with a stride of 2 and a kernel size of 4 × 4. The output dimensions of each convolutional layer are as follows: 1 × 64 × 64, 128 × 32 × 32, 256 × 16 × 16, 512 × 8 × 8, 1024 × 4 × 4. The resultant output is remodeled in order to conform to a predetermined shape, subsequently being inputted into a fully interconnected layer, in which the ensuing output value is utilized as the basis for the discriminator’s decision.

The batch size is 64, the learning rate is 0.001, and the model is trained for 50 epochs using the Leaky ReLU activation function. The Adam optimizer is used with a β1 value of 0.5.

In the context of the Transformer diagnostic model, the parameter configuration used in the experiment is as follows: a patch size of 4 × 4, 2 encoder layers, a token dimension of 96, and 12 attention heads. The maximum number of iterations that can occur during the training phase of the model has been set to 50.

## 4. Result Analysis and Discussion

In Section 3, after identifying the fault types for each analog circuit test case, data were collected, and model parameters were set. Subsequently, fault diagnosis tests were conducted on the three circuits to validate the effectiveness of the proposed data generation and fault diagnosis methods. This section will primarily focus on presenting and analyzing the experimental results.

### 4.1. Diagnostic Results of the Proposed Method

After the fault is determined, the data are collected, and the model parameters are set, subsequent fault diagnosis tests are conducted on three circuits to validate the efficacy of the proposed data generation and fault diagnosis methodologies. Specifically, five distinct tests were conducted for the single fault and compound fault configurations of the aforementioned circuits to prevent the occurrence of arbitrary test outcomes. The sample size for each fault category in each test instance was set at 50. In addition, it is essential to note that in the training process of the Transformer for fault diagnosis, the actual sample size and the generated sample size were set at 150, respectively. Table 4 shows the specific classification results of each fault category of CUT1 single fault in a single test. For a single CUT1 circuit fault case, three indicators were calculated for each fault category, namely Precision, Recall, and Specificity. Among them, the Precision index measures the ratio of accurately predicted positive samples, while the Recall index evaluates the proportion of positively predicted samples, indicating the degree to which positive samples are identified. The Specificity index refers to the ratio of correctly identified negative samples out of all negative samples.

**Figure 14:**
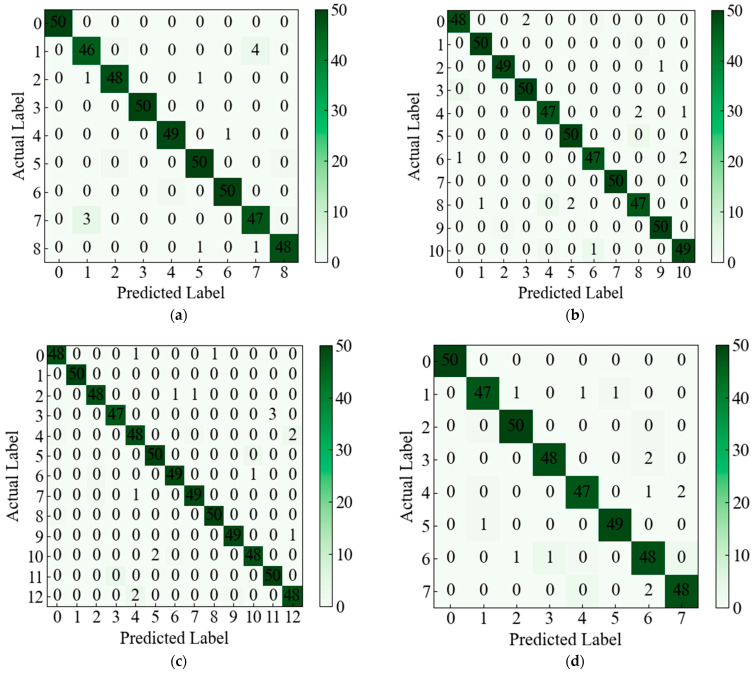
Confusion matrix of diagnostic results of the proposed method in four cases. (**a**) CUT1 single fault case. (**b**) CUT2 single fault. (**c**) CUT3 single fault. (**d**) CUT1 compound fault.

As is evident from Table 4, for the fault-free category F0, all three index values are all 1, indicating that all samples of this category are correctly identified. According to the values of the three indexes, F1 and F7 fault categories have the most serious misclassification. In addition, the confusion matrix of the single fault diagnosis result of CUT1, CUT2, and CUT3 and the composite fault diagnosis result of CUT1 is shown in Figure 14.

As displayed in Figure 14, in the three cases of single fault diagnosis, among the 450 test samples of CUT1 shown in Figure 14a, all the normal samples were correctly classified, and a total of 11 fault samples were misclassified. In the test samples of CUT2, as displayed in Figure 14b, all 13 samples were mismarked. A total of 16 samples were mismarked in CUT3, as shown in Figure 14c. As shown in Figure 14d, a total of 13 samples of CUT1 compound fault were misclassified. In the figure, the overall classification accuracy of these four cases has reached more than 96%. It can be observed that the proffered methodology has exhibited a commendable classification efficacy, both in instances of single faults and compound faults.

### 4.2. Comparison Experiments

To analyze the superiority of the proffered scheme, a comparative experiment is set up in this paper. A total of four comparison methods were set. The first comparison method involves transforming the time domain signal generated by the circuit output into a two-dimensional signal with the aim of examining and diagnosing fault signals from a different perspective. As a comparison with CWT, the subsequent DCGAN generation module and Transformer feature mining and classification module remain unchanged. This comparison method is called TGT for short. The second comparison method is to assess the merits of the proposed data generation method; it would be viable to compare the modified version with the original GAN by utilizing the DCGAN data generation module. This comparison method is called CGT for short. In addition, to examine the benefits of incorporating Transformer modules, it is necessary to analyze their various advantages so that the signal mining modules of two-dimensional CNN and ResNet 35, which have advantages in mining two-dimensional signals, are selected for comparison with the Transformer module. The third and fourth comparison methods are referred to as CGC and CGR, respectively.

**Figure 15:**
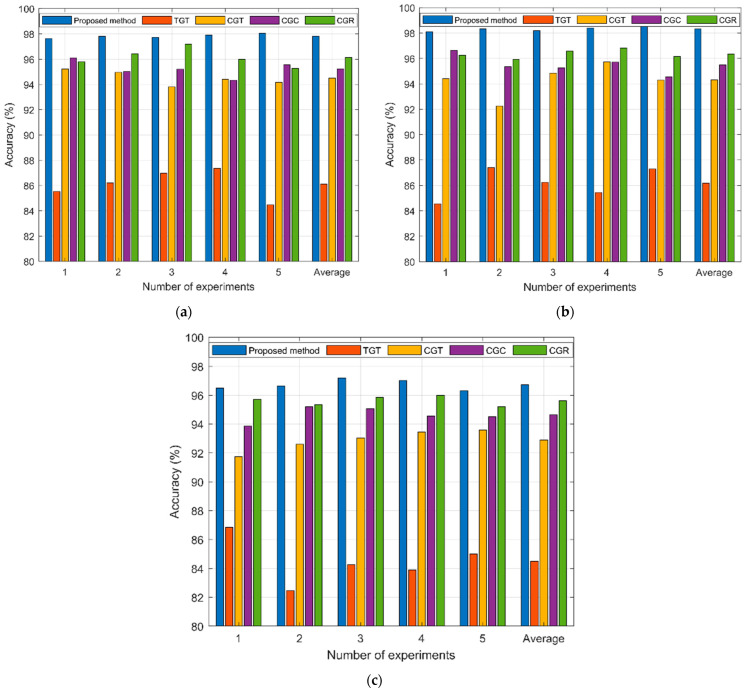
The diagnostic results of the proposed method and the four comparison methods in 5 tests. (**a**) CUT1 single fault; (**b**) CUT2 single fault; (**c**) CUT3 compound fault.

It is imperative to evaluate the effectiveness of the proposed approach and compare it with the four benchmark techniques in order to assess its performance in the given task. And three cases were tested, namely CUT1 single fault, CUT2 single fault, and CUT3 compound fault. First, five tests were carried out in each of the three cases, and the accuracy of each test is shown in the bar chart in Figure 15.

As displayed in Figure 15, for the three test cases, the performance of the proposed method is the best compared with the four comparison methods. Taking CUT1 single fault as an example, the proposed method demonstrates an average accuracy of 97.8% in five distinct tests, attaining a remarkable level of performance, while the diagnostic accuracy of the three comparison methods is 86.2 (TGT), 94.2 (CGT), 95.2 (CGC), and 96.1 (CGR), respectively. In addition, for the three test cases as a whole, among the four comparison methods, comparison method 4 has the best performance, followed by comparison method 3 and 2, while comparison method 1 has the worst performance. The feature extraction of the circuit output signal through wavelet transformation exhibits a notable positive influence on the subsequent classification process. DCGAN produces samples that are more closely similar to real data, surpassing GAN in accuracy. Transformer is better at image feature mining and classification than 2D CNN and ResNet 35.

**Figure 16:**
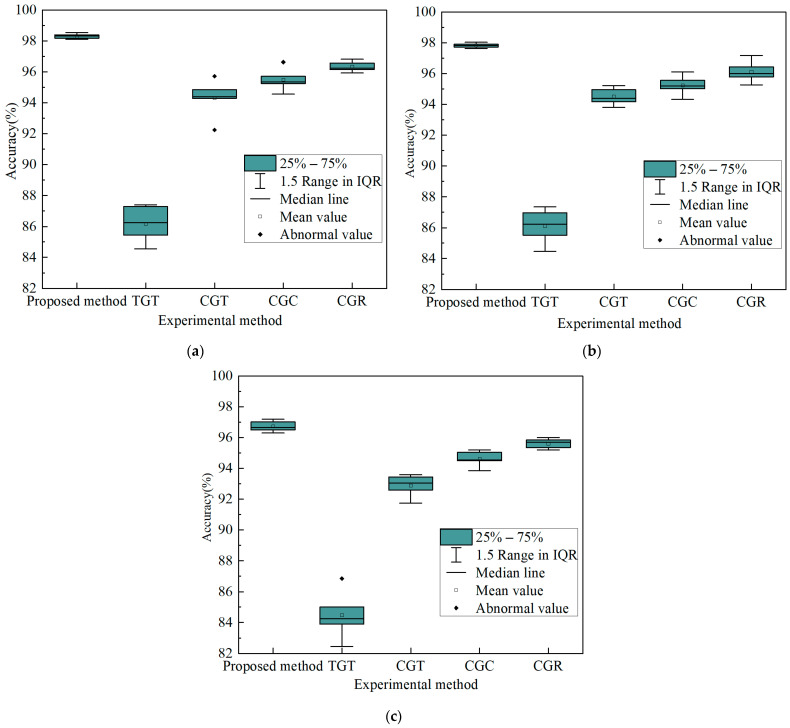
Box diagram of diagnosis results of the proposed method and the four comparison methods. (**a**) CUT1 single fault. (**b**) CUT2 single fault. (**c**) CUT3 compound fault.

In addition, using the three test cases mentioned above as examples, Figure 16 displays box plots for the proposed method and four comparison methods across five experiments.

As shown in Figure 16, the diagnostic accuracy of the proposed method is superior to that of the four comparison methods for the three test cases. In addition, in terms of the distance from 1/4 quantile to 3/4 quantile, in the three test cases, compared to the four evaluated methods, the proposed approach appears to be more compact. In the comparison methods, the first comparison method has the largest distance from the first quartile to the third quartile, while the other three comparison methods are relatively closer. Therefore, it is evident that the proposed method displays stable and robust performance, while the robustness of the comparison method 1 is the worst.

### 4.3. Small Sample Dataset Testing

For the purpose of evaluating the impact of a small sample size on the data quality generated by the DCGAN model and then determining the appropriate sample size suitable for the generated data, a total of five different training sets were set up, and the number of real and generated samples is diverse in each combination, but for each fault class in any circuit case, the total number of training set samples is 300. In this paper, the CUT2 compound fault is used as an illustrative example, and the specific settings of the dataset are shown in Table 5, which also gives the average accuracy results of five tests of the mentioned method.

Table 5 reveals a rising trend in the average accuracy with an augmented number of real samples in the training set and a reduced number of artificially generated samples. Given the quantities of real samples and artificially generated samples, which are both 150, the average attained level of precision is 96.65%. However, moving forward in the composition of this training dataset, the rate of improvement in diagnostic performance of the proposed algorithm noticeably decreases. Therefore, considering the difficulty of obtaining the real sample size, it is plausible to assume that the proposed technique has achieved satisfactory diagnostic performance when the number of both real and generated samples reaches 150.

### 4.4. Research Outcomes and Limitations of the Proposed Method

In this study, the problem of overfitting easily caused by insufficient data in analog circuits is solved, and automatic feature extraction, sample expansion, and high-precision fault classification can be realized in the CWT-DCGAN-Transformer model. How to automatically optimize hyper-parameters such as the learning rate and network structure of the model is still insufficient.

## 5. Conclusions

To address the challenge of limited fault samples in electronic equipment, this paper has developed a small-sample fault diagnosis approach for simulated circuits based on CWT-DCGAN-Transformer, achieving two significant breakthroughs.

(i) In scenarios with a limited number of real fault samples, the proposed approach demonstrates satisfactory performance. For instance, in cases where the real sample size is only 150, the diagnostic accuracy of a test case exceeds 96%;
(ii) The proposed approach eliminates the need for the manual design of fault features. Through the joint processing of continuous wavelet transformation and the Transformer model, it autonomously explores time–frequency fault features and achieves self-attention and classification of these features. This leads to efficient diagnostic performance.

On one hand, this work offers a solution to the challenging problem of small-sample fault diagnosis in practical engineering. On the other hand, it explores the potential of Transformer models in the field of fault diagnosis. In the future, it is of paramount importance to explore how to automatically optimize the different modules and networks within the proposed approach.

## Figures and Tables

**Table 1:** Single fault of Sallen–Key bandpass filter circuit.

Fault Class	Nominal Value	Fault Code	Fault Value
NF	-	F0	-
C1↑	5.00 nF	F1	[5.50 nF, 6.50 nF]
C1↓	5.00 nF	F2	[3.50 nF, 4.50 nF]
C2↑	5.00 nF	F3	[5.50 nF, 6.50 nF]
C2↓	5.00 nF	F4	[3.50 nF, 4.50 nF]
R2↑	1.00 kΩ	F5	[1.05 kΩ, 1.30 kΩ]
R2↓	1.00 kΩ	F6	[0.70 kΩ, 0.95 kΩ]
R3↑	2.00 kΩ	F7	[1.05 kΩ, 1.30 kΩ]
R3↓	2.00 kΩ	F8	[0.70 kΩ, 0.95 kΩ]

**Table 2:** Single fault of Thomas filter circuit.

Fault Class	Nominal Value	Fault Code	Fault Value
NF	-	F0	-
C1↑	10.00 nF	F1	[11.00 nF, 13.00 nF]
C1↓	10.00 nF	F2	[7.00 nF, 9.00 nF]
C2↑	10.00 nF	F3	[11.00 nF, 13.00 nF]
C2↓	10.00 nF	F4	[7.00 nF, 9.00 nF]
R3↑	2.00 kΩ	F5	[2.10 kΩ, 2.60 kΩ]
R3↓	2.00 kΩ	F6	[1.40 kΩ, 1.90 kΩ]
R4↑	2.00 kΩ	F7	[2.10 kΩ, 2.60 kΩ]
R4↓	2.00 kΩ	F8	[1.40 kΩ, 1.90 kΩ]
R5↑	2.00 kΩ	F9	[2.10 kΩ, 2.60 kΩ]
R5↓	2.00 kΩ	F10	[1.40 kΩ, 1.90 kΩ]

**Table 3:** Compound faults of Thomas filter circuit.

Fault Class	Nominal Value	Fault Code	Fault Value
NF	-	F0	-
C1↑C2↑	10.00 nF, 10.00 nF	F1	[11.00 nF, 13.00 nF], [11.00 nF, 13.00 nF]
C1↓C2↓	10.00 nF, 10.00 nF	F2	[7.00 nF, 9.00 nF], [7.00 nF, 9.00 nF]
C2↑R3↑	10.00 nF, 2.00 kΩ	F3	[11.00 nF, 13.00 nF], [2.10 kΩ, 2.60 kΩ]
C2↓R3↑	10.00 nF, 2.00 kΩ	F4	[7.00 nF, 9.00 nF], [2.10 kΩ, 2.60 kΩ]
R3↑R5↑	2.00 kΩ, 2.00 kΩ	F5	[2.10 kΩ, 2.60 kΩ], [2.10 kΩ, 2.60 kΩ]
R3↑R5↑C1↑	2.00 kΩ, 2.00 kΩ, 10.00 nF	F6	[2.10 kΩ, 2.60 kΩ], [2.10 kΩ, 2.60 kΩ], [11.00 nF, 13.00 nF]
R3↑C1↑C2↓	2.00 kΩ, 10.00 nF, 10.00 nF	F7	[2.10 kΩ, 2.60 kΩ], [11.00 nF, 13.00 nF], [7.00 nF, 9.00 nF]

**Table 4:** Fault diagnosis results of CUT1 single fault test.

Fault Class	Fault Code	Precision	Recall	Specificity
F0	NF	1	1	1
F1	C1↑	0.92	0.92	0.99
F2	C1↓	1	0.96	1
F3	C2↑	1	1	1
F4	C2↓	1	0.98	1
F5	R2↑	0.96	1	0.995
F6	R2↓	0.98	1	0.9975
F7	R3↑	0.90	0.94	0.9875
F8	R3↓	1	0.96	1

**Table 5:** Comparison results of the impact of different training set compositions on the diagnosis of the proposed method.

Different Composition of Training Sets Fault Code		Average Accuracy (%)
Real Sample Size	Generated Sample Size	
50	250	82.30 (1646/2000)
100	200	88.50 (1770/2000)
150	150	96.65 (1933/2000)
200	100	97.40 (1948/2000)
250	50	98.15 (1963/2000)

## Data Availability

The data presented in this study are available in this article.
